# Automatic assessment of knee osteoarthritis severity in portable devices based on deep learning

**DOI:** 10.1186/s13018-022-03429-2

**Published:** 2022-12-14

**Authors:** Jianfeng Yang, Quanbo Ji, Ming Ni, Guoqiang Zhang, Yan Wang

**Affiliations:** 1 Medical School of Chinese PLA, Beijing, 100853 China; 2 Department of Orthopedics, The First Medical Center, Chinese People’s Liberation Army General Hospital, Fuxing Road, Haidian District, Beijing, 100048 China; 3 Senior Department of Orthopedics, The Fourth Medical Center of PLA General Hospital, Beijing, 100048 China

**Keywords:** Deep learning, Knee osteoarthritis, Kellgren–Lawrence, Artificial intelligence

## Abstract

**Background:**

For knee osteoarthritis, the commonly used radiology severity criteria Kellgren–Lawrence lead to variability among surgeons. Most existing diagnosis models require preprocessed radiographs and specific equipment.

**Methods:**

All enrolled patients diagnosed with KOA who met the criteria were obtained from **** Hospital. This study included 2579 images shot from posterior–anterior X-rays of 2,378 patients. We used RefineDet to train and validate this deep learning-based diagnostic model. After developing the model, 823 images of 697 patients were enrolled as the test set. The whole test set was assessed by up to 5 surgeons and this diagnostic model. To evaluate the model’s performance we compared the results of the model with the KOA severity diagnoses of surgeons based on K-L scales.

**Results:**

Compared to the diagnoses of surgeons, the model achieved an overall accuracy of 0.977. Its sensitivity (recall) for K-L 0 to 4 was 1.0, 0.972, 0.979, 0.983 and 0.989, respectively; for these diagnoses, the specificity of this model was 0.992, 0.997, 0.994, 0.991 and 0.995. The precision and F1-score were 0.5 and 0.667 for K-L 0, 0.914 and 0.930 for K-L 1, 0.978 and 0.971 for K-L 2, 0.981 and 0.974 for K-L 3, and 0.988 and 0.985 for K-L 4, respectively. All K-L scales perform AUC > 0.90. The quadratic weighted Kappa coefficient between the diagnostic model and surgeons was 0.815 (*P* < 0.01, 95% CI 0.727–0.903). The performance of the model is comparable to the clinical diagnosis of KOA. This model improved the efficiency and avoided cumbersome image preprocessing.

**Conclusion:**

The deep learning-based diagnostic model can be used to assess the severity of KOA in portable devices according to the Kellgren–Lawrence scale. On the premise of improving diagnostic efficiency, the results are highly reliable and reproducible.

## Introduction

Knee osteoarthritis (KOA) is a degenerative joint disease with a prevalence ranging from 4 to 12% [1–3]. Currently, for orthopaedic surgeons, knee weight-bearing standing X-ray radiographs as a standard method for evaluating KOA remain the most common radiology examination method due to their safety, popularity and low cost [4–6]. Currently, accurate KOA diagnosis and assessment are highly based on radiographic evidence [7, 8].

Currently, the Kellgren–Lawrence scale (K-L) is most commonly used to diagnose and determine the severity of KOA based on joint space narrowing, osteophytes, sclerosis, and definite bony deformities on X-rays in radiology examinations [9, 10]. However, the classification criteria of the K-L scales are subjective [11]. In clinical use, different doctors or the same doctor at different times may often obtain similar results rather than identical results on the same X-ray. Many clinical studies involving KOA diagnosis have ensured reliability by increasing the number of repeated diagnoses [12–16]. Moreover, the total numbers of X-ray examinations are much higher in large hospitals, which is a heavy burden on radiologists and surgeons. Therefore, many rapid diagnosis and assessment models have been developed in collaboration with image analysis. The models can identify images via digital processing techniques to make the artificial intelligence process more accurate and cost-effective [13]. The technology includes knee joint recognition and image processing based on deep learning [14]. Swiecicki et al. [15] developed a diagnostic model based on the two-stage Faster R-CNN model to assess the severity of KOA from both posterior–anterior (PA) and lateral (LAT) views. Tiulpin et al. [16] also developed a diagnostic model based on the ResNet34 model to detect KOA from original PA views of knees. Similarly, Norman et al. [17] developed a KOA diagnostic model based on DenseNet, which uses the feature more effectively in deep technology. Current studies show that the existing diagnostic models can achieve satisfactory accuracy [15–17]. However, those models rely on preprocessed, highly optimized digital images in specific software and hardware, which may not be feasible in most clinical scenarios and affects the actual use value in some ways [18–23]. We developed a fast, easy-to-use model based on portable devices to facilitate the diagnosis of KOA in clinical situations.

This retrospective study aimed to develop an algorithm-based diagnostic model for KOA showing on unpreprocessed radiographs in portable devices. The X-ray-based KOA was evaluated by surgeons and diagnostic models, and the results were compared. We hypothesized that this model can achieve the same or similar accuracy as surgeons.

## Materials and methods

The study was approved by the Institutional Review Board of the hospital involved. Informed consent was obtained from all participants. All methods were performed in accordance with the relevant guidelines and regulations.

We retrospectively collected radiographs of patients who underwent radiographic examination at the **** Hospital from January 2020 to January 2021 (each patient may have multiple radiographs). All radiographs were taken using uDR 780i Pro Fully Automatic Celling-mounted DR (UNITED IMAGING, Shanghai, China). Patients meeting the following criteria were included: (1) age ≥ 40, (2) a KOA diagnosis established based on the Chinese Guidelines for the Diagnosis and Treatment of Osteoarthritis (2019 edition) [24], (3) combined with pain, limited movement or other symptoms, (4) unilateral or bilateral weight-bearing standing posterior–anterior (PA) X-rays of knee joints, and (5) previous unilateral knee surgery history or no surgery history of both knees. Patients meeting the following criteria were excluded: (1) nonweight-bearing standing position or lateral X-rays of knee joints, (2) diagnosed inflammatory arthropathies (such as comorbidity such as rheumatoid arthritis, ankylosing spondylitis), (3) severe knee joint deformity, (4) other comorbidities that may cause knee joint deformity (such as arthritis of haemophilia, enteropathic arthropathy or traumatic osteoarthritis), (5) history of intra-articular fracture or fractures around the knee, (6) X-ray shows fusion on knee joint, (7) diagnosed infectious arthritis or postoperative joint infection, (8) implants of both knee joints shown in a radiograph (such as internal fixation or knee prosthesis), (9) incorrect posture (such as rotation or flexion of the lower limbs); and (10) poor image quality.

After reviewing the 2,579 included PA X-rays from 2,378 patients, we divided these radiographs into training (1,598 radiographs), validation (158 radiographs) and test sets (823 radiographs) (as shown in Table 1). Each X-ray in the training set and validation set was scaled by skilled radiologists and orthopaedic surgeons based on K-L scales (K-L 0: No evidence of KOA; K-L 1: The possibility of joint space narrowing and osteophyte formation; K-L 2: Definite osteophyte formation and possible joint space narrowing; K-L 3: Multiple osteophytes, definite joint space narrowing, sclerosis, and possibly bone deformity; K-L 4: End-stage KOA marked by severe sclerosis, joint space narrowing, and large osteophytes).

**Table 1:** Dataset split of included patients

Classification	Training set	Validation set	Test set
Patients	1532	149	697
Radiographs	1598	158	823
Right knees	1337	141	495
Left knees	1162	122	446
Total knees	2499	263	941
Implants	267	21	96
TKA	158	12	59
UKA	86	4	24
PFA	8	1	3
E–F	2	1	3
I-F	13	3	7
*K-L scales*			
K-L 0	49	1	3
K-L 1	214	10	59
K-L 2	237	26	146
K-L 3	1002	115	379
K-L 4	997	111	354
Total	2499	263	941

*TKA* Total knee arthroplasty; *UKA* Unicompartmental knee arthroplasty; *I-F* Internal fixation; *E–F* External fixation; *PFA* Patellofemoral arthroplasty; *K-L* Kellgren–Lawrence scales

### Radiograph preprocessing

All radiographs were captured using mobile phones (iPhone 8 Plus, Apple Inc.) During the shoot, in the case of an indoor light source, the phone was fixed on a tripod, 40 cm away from the radiographs, flush with the centre of the radiographs. All photographs were stored in JPG format as 4000 × 3000 pixels. For the training set and validation set, cropped localization ground truths around the knee were made manually by skilled radiologists and orthopaedic surgeons and implemented in MATLAB (Mathworks, Natick, MA). Those are resized to 512 × 512 pixels using a bilinear interpolation algorithm and then normalized by the z score method with statistical mean pixel intensities and variances.

### Model architecture

**Fig. 1:**
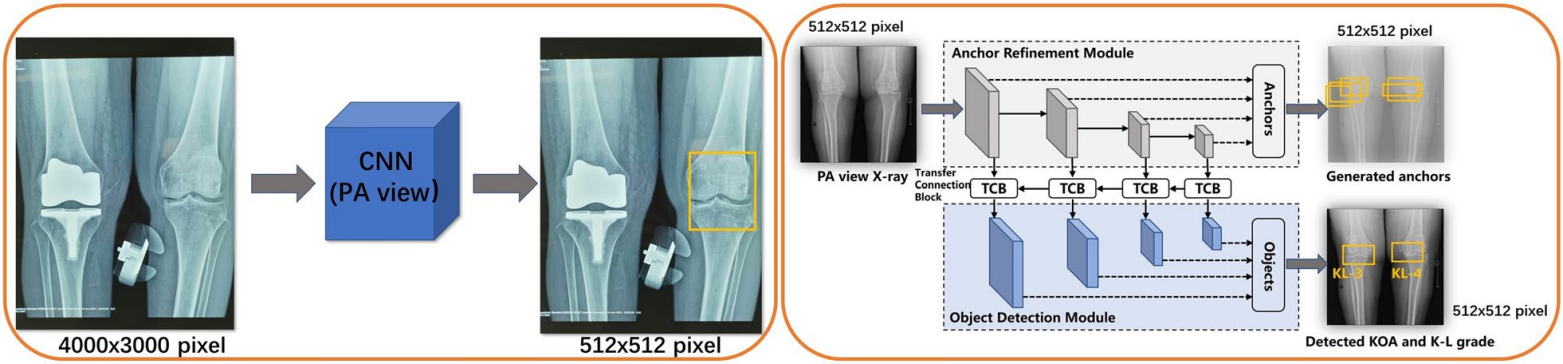
Pipeline of RefineDet Model for K-L Classification *CNN* Convolutional Neural Network; *PA* Posterior–anterior view standing bearing X-ray; *TCB* Transfer connection block; *OA* Osteoarthritis; *K-L* Kellgren–Lawrence

We formulate the KOA severity assessment to a detection task: to localize the knee joint while classifying the K-L scale based on a prevalent detection model RefineDet [25] (the proposed model is shown in Fig. 1). It consists of two connected modules, an anchor refinement module (ARM) and an object detection module (ODM). Specifically, the ARM is designed to localize the knee joint area with bounding boxes, i.e., anchors and generate the candidate anchors on the left/right knees for ODM. The ODM takes the candidate anchors as the input to further refine the localizations and sizes of anchors and predict the K-L scale of the corresponding knee joint. In addition, the transfer connection block (TCB) is introduced to convert the features from ARM to ODM at different scales and fuse context information from high-level features to improve detection accuracy. The ARM is trained with anchor binary classification loss and anchor bounding box regression loss, while the ODM is trained with K-L scale classification loss and knee joint bounding box regression loss.

In object detection, range of interest (ROI) placement is primarily located through the ARM module and ODM module in RefineDet. The ARM module retrieved proposals through 4 stages of the CNN block as a candidate set of ROIs. Each proposal predicts the corresponding bounding box coordinates and judges whether it is an ROI. These proposals provided the initial information for the subsequent detection of the ODM module. The ODM module integrated information from different stages, further regressed the coordinates of the ROI to improve positioning accuracy, and classified the targets in the ROI. Since the model can predict multiple proposals, we conducted postprocessing of Non-Maximal Suppression (NMS)^36^ according to the confidence of different proposals with the IoU threshold of 0.5, and finally obtained one ROI for a knee joint.

The proposed model is implemented using PyTorch and trained on a machine with 4 Nvidia P100 GPUs. The parameters of the network are initialized with the pretrained model from the large public dataset ImageNet [26]. In addition, training datasets are augmented by several methods followed [27]. During training, the network is optimized by Adam [28] with the learning rate first warmed from 2e-2 to 1e-1 for 1e4 iterations and then decreased to 1e-2, 1e-2 and 1e-3 at the 3e4, 3.5e4 and 4e4 epochs. The momentum is set to 0.9, and the weight decay is set to 5e-4. The overall optimization is carried out for 4e5 iterations with a batch size of 128. In actual use, the model was used to evaluate both medial and lateral compartments of knee joints. The diagnosis of the more severe compartment was used as the diagnosis of KOA in a knee joint according to the K-L grade. For example, as the schematic diagram of the RefineDet model shown in Fig. 1, the left knee was diagnosed as K-L 4 mainly due to more severe stenosis of the medial compartment. The right knee was diagnosed as K-L 3 due to more osteophytes in the medial compartment and similar stenosis of both compartments.

### Surgeons’ evaluation

**Fig. 2:**
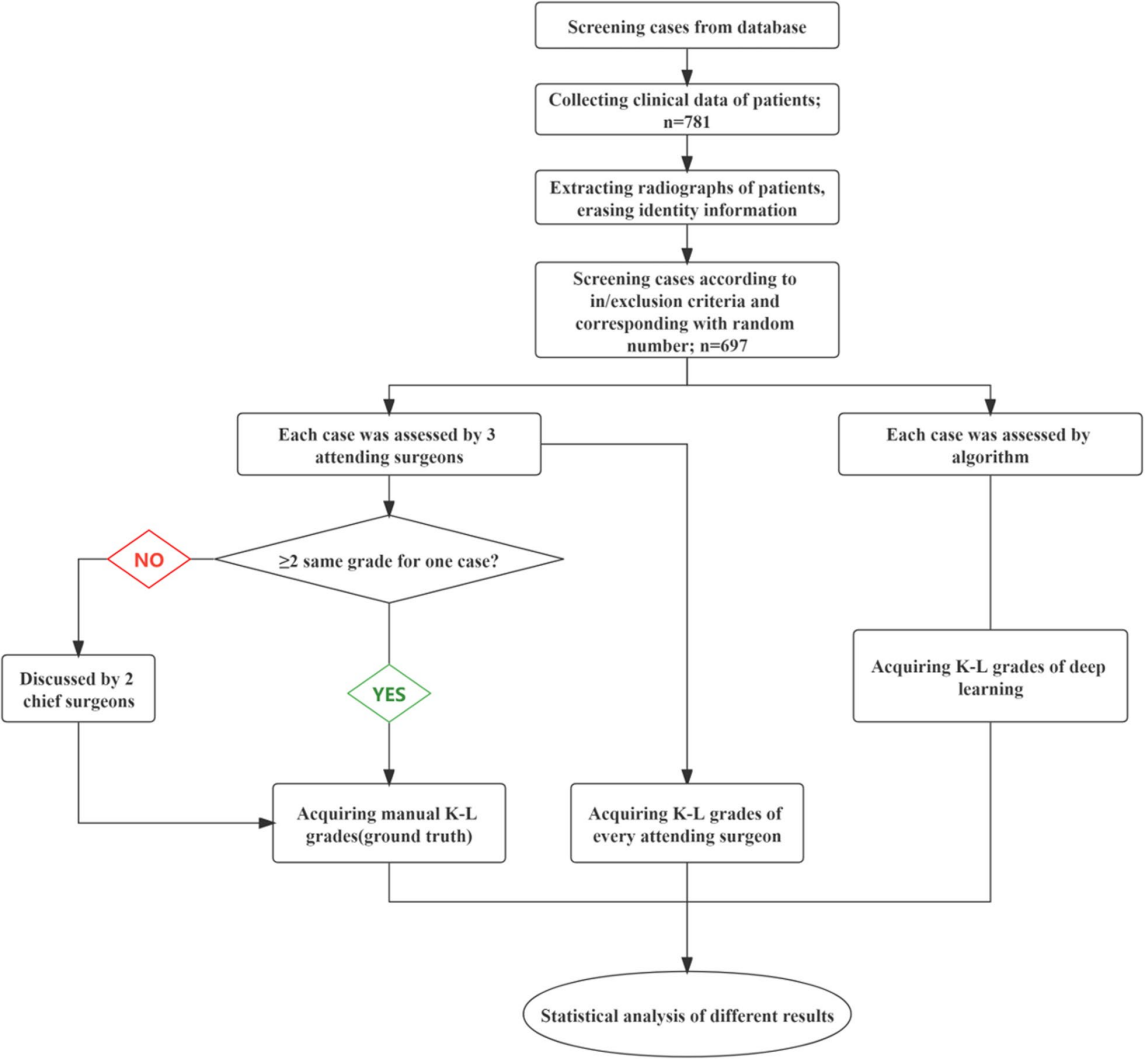
Experimental flowchart for test set *K-L* Kellgren–Lawrence

For the test set, the ground outcomes were first evaluated by up to 5 members of our research team. Based on the K-L scales, three senior orthopaedic surgeons assessed the severity of KOA after the identity information of patients was removed. For each photograph, when there are two or three of the same K-L scale, it is used as the ground truth. The inconsistent or doubted evaluations were discussed and determined by two chief surgeons (the flowchart is shown in Fig. 2).

### Statistical analysis

To assess the performance of the diagnostic model, we adopted accuracy, precision, recall, AUC, sensitivity and specificity as indicators. We also used the quadratic weighted Kappa coefficient to verify the consistency among the model, manual summary (results were put together by all surgeons), and each senior orthopaedic surgeon [29]. To make it more intuitive, we adopted a confusion matrix to visualize the results. The model was developed and trained using PyTorch (v. 1.4). The data were analysed using R (4.0.0) and SPSS Version 25.0.0.2 (SPSS, Inc., Chicago, Ill.).

## Results

We included 1,598 PA X-rays in the training set, 158 PA X-rays in the validation set and 823 in the test set (as shown in Table 1). The most common KOA severities of patients in this study were K-L 3 (1,481 knee joints) and K-L 4 (1,449 knee joints), which is similar to the results that we observed in the clinic. For the test set used to assess the performance of this model, as shown in Table 2, the mean age of the patients was 61.7 ± 11.2 yrs (40–87 yrs old), 12.2% (n = 85) had a unilateral knee surgery history, and a total of 352 patients had comorbidities (one patient may have multiple comorbidities). The diagnostic model identified 95.7% of all knee joints (901 of 941, and no implants included) in the test set.

**Table 2:** Characteristics of patients in test set

Variable	Overall (n = 697)
Male(no.[%])	221 (31.8%)
Female(no.[%])	476 (68.3%)
Age (years)	61.7 ± 11.2 (40–87)
Comorbidity	352
Hypertension	221
Diabetes	104
Hyperlipemia	128
CHD	152
Osteoporosis	193
Mental disorder	38
Surgery history(no.[%])	96
unilateral UKA	24
unilateral TKA	59
unilateral HTO	5
unilateral PFA	3
unilateral bone fracture	5

*TKA* total knee arthroplasty; *UKA* Unicompartmental knee arthroplasty; *I-F* Internal fixation; *E–F* External fixation; *PFA* Patellofemoral arthroplasty; *KOA* Knee osteoarthritis; *CHD* Coronary heart disease

For the assessment of KOA-based K-L scales (a five-category classification task), in the validation set, the model’s sensitivity (also called recall) and specificity for assessing KOA severity were 1.0 and 0.992 for K-L 0, 0.972 and 0.997 for K-L 1, 0.979 and 0.994 for K-L 2, 0.983 and 0.991 for K-L 3, and 0.989 and 0.995 for K-L 4. The corresponding sensitivity (recall) and specificity in the test set were 1.0 and 0.998 for K-L 0, 0.946 and 0.994 for K-L 1, 0.971 and 0.996 for K-L 2, 0.978 and 0.987 for K-L 3, and 0.982 and 0.993 for K-L 4, respectively (as shown in Table 3). For each K-L scale, the sensitivity (recall) and specificity showed that the model indicated the proportion of true prediction samples in patients diagnosed with this scale or false prediction samples in non-this-scale patients (Table 4).

**Table 3:** Precision, Recall, F1 score and AUC of test set in each scale of knee osteoarthritis

K-L scale	Knee(n = 901)	Precision	Recall	F1 score	AUC (95% CI)
K-L 0	2	0.5	1	0.667	0.999
					(0.997–1.0)
K-L 1	56	0.914	0.946	0.93	0.97
					(0.936–1.0)
K-L 2	138	0.978	0.971	0.971	0.983
					(0.966–0.999)
K-L 3	364	0.981	0.978	0.974	0.981
					(0.970–0.992)
K-L 4	341	0.988	0.982	0.985	0.988
					(0.979–0.997)

*K-L* Kellgren–Lawrence; *AUC* Area under curve

**Table 4:** Sensitivity and specificity of validation set and test set in each scale of knee osteoarthritis

	Validation set		Test set	
K-L scale	Sensitivity	Specificity	Sensitivity	Specificity
K-L 0	1	0.992	1	0.998
K-L 1	0.972	0.997	0.946	0.994
K-L 2	0.979	0.994	0.971	0.996
K-L 3	0.983	0.991	0.978	0.987
K-L 4	0.989	0.995	0.982	0.993

*K-L* Kellgren–Lawrence

Assessing the performance of the model in the test set achieved an accuracy of 95.7%, which indicates the proportion of K-L scales predicted correctly in all included samples. This model made the entire assessment pipeline just under 5 s for a given unpreprocessed image. The precision was 0.5 (AUC = 0.999 *P* = 0.015, 95% CI 0.997–1.0) for K-L 0, 0.914 (AUC = 0.970 *P* < 0.01, 95% CI 0.936–1.0) for K-L 1, 0.978 (AUC = 0.982 *P* < 0.01, 95% CI 0.966–0.999) for K-L 2, 0.981 (AUC = 0.981 *P* < 0.01, 95% CI 0.970–0.992) for K-L 3, 0.988 (AUC = 0.988 *P* < 0.01, 95% CI 0.979–0.997) for K-L 4, which shows the proportion of images of a given K-L scale in the predicted images of this scale for K-L 0 to 4. The F1-score, combining precision and recall to avoid imbalances of different K-L scales in included images, was 0.667, 0.930, 0.971, 0.984 and 0.985 for K-L 0 to 4, respectively.

**Fig. 3:**
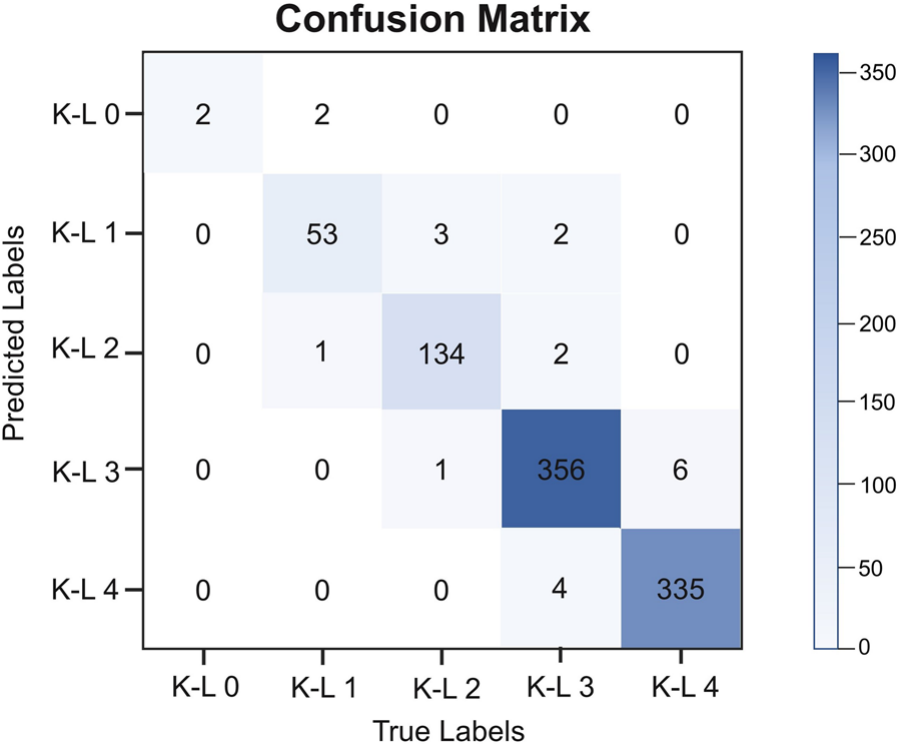
Confusion matrix of K-L scales Confusion matrix shows the diagnosis results of model and summary by surgeons. True labels (diagnosed by surgeons) are the columns and the predicted labels (diagnosed by model) are the rows. The diagonal represents the number of correct predictions for each K-L scale. The total number of subjects in each group can be obtained by summing that respective column. Darker squares represent a higher percentage of that group classified for a predicted label) *K-L* Kellgren–Lawrence

**Fig. 4:**
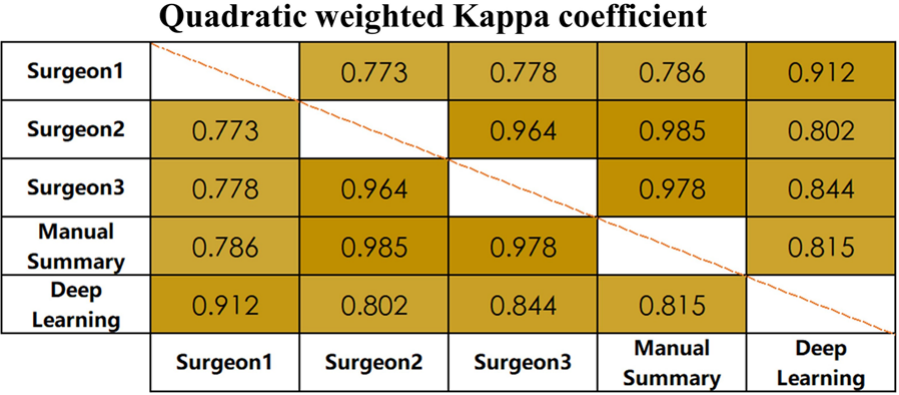
Quadratic weighted Kappa coefficient Quadratic weighted Kappa coefficient shows the consistency between all pairs Surgeon: Diagnosis results of each surgeon Deep Learning: Diagnosis results of diagnostic model Manual Summary: Summary of diagnosis results of all surgeons *K-L* Kellgren–Lawrence (*P* < 0.01)

The confusion matrix is shown in Fig. 3, which recorded the samples assessed by the model and surgeons in the test set according to K-L scales. The diagonal represents the number of consistent diagnoses between surgeons and the model for each scale (2, 53, 134, 356 and 335, respectively). The quadratic weighted Kappa coefficient between the surgeons’ summary and diagnostic model was 0.815 (*P* < 0.01, 0.727–0.903). The average quadratic weighted Kappa coefficient between the model and each surgeon was 0.853 (*P* < 0.01, 95% CI 0.769–0.936) (as shown in Fig. 4), which shows the consistency between the model and clinical diagnosis.

## Discussion

Compared to similar studies [12, 15–19] this study further demonstrates the potential of implementation to aid in the diagnosis and even acceptably classifying KOA. This model achieved an accuracy of 95.7%, which was approximate to or higher than those of similar studies. For given photographs showing different severities of KOA, this model can achieve high-accuracy diagnosis based on K-L scales and avoid time consumption. The sensitivity and specificity of the test set were similar to those of the validation set on different K-L scales (as shown in Table 3). In K-L 0, the precision was 0.5. In addition to 2 correct diagnoses, another 2 middle-aged women (54 years and 58 years) were misdiagnosed as K-L 0 in the model because each of their PA X-rays showed mild osteoporosis on one knee, and surgeons diagnosed K-L 1 as potentially KOA. Some similar patterns were seen in patients with K-L 1, but for those diagnosed as K-L 0 or K-L 1, intervention is usually not needed. Therefore, the results do not mislead clinical treatment. For K-L 3, the specificity was 0.987. After analysing these patients, we found that some patients with advanced age, poor mobility and other conditions were diagnosed as K-L 4 by surgeons, but their PA X-rays showing were not completely consistent with the symptoms, so some of them were misdiagnosed as K-L 3 by this model. In fact, the clinical treatment of such patients is basically the same as that of K-L 4 patients diagnosed by the model, so it does not largely impact the actual use. In Fig. 3, the confusion matrix shows that the diagnosis of KOA severity is consistent between surgeons and the model in the vast majority of included cases. In Fig. 4, the quadratic weighted Kappa coefficient between the manual summary and this model was generally excellent (0.815 > 0.8), which indicates the reliability of this model. The sensitivity and specificity between the validation and test sets also suggest that the results of diagnoses are reliable and reproducible. In the actual test process, the efficiency of using the model for diagnosis is indeed significantly higher than that of repeated diagnoses by surgeons.

Currently, in many studies involving radiology diagnosis of KOA, researchers are increasingly using an increasing number of repeated diagnoses for one X-ray to assure reliable results due to limitations of the K-L scales. Benefiting from the rapid growth of artificial intelligence in clinical use, algorithm-assisted diagnosis can effectively acquire highly reliable and repeatable results in these situations. However, the existing diagnostic models are mostly based on high-quality images integrated into data centres [16, 17, 23, 30, 31]. Images storage and use of models are not convenient, most need preprocessing and particular equipment. Typical photographs taken by mobile phones are rarely used due to suboptimal camera angles or lighting or other factors. The major difference between our study and those mentioned above is that we allowed the RefineDet model to diagnosis in PA X-rays without preprocessing. Unlike the study by Swiecicki et al. [15] using a two-stage Faster R-CNN model, we used a one-stage RefineDet model for KOA severity on PA X-rays. From the results, our diagnostic model was more accurate (Faster R-CNN 70.9% vs. RefineDet 95.7%). And from the architecture of the model, one-stage methods were less computing and more efficient than two-stage methods. After the actual use, we speculated that one-stage methods may be more advantageous in high-volume clinical data application scenarios such as outpatient clinics. And Guan et al. [31] using a one-stage YOLO model for joint cropping, validated the one-stage model to be also reliable in predicting radiographic medial joint space loss within a 48-month follow-up period. In contrast to other studies, RefineDet model achieved higher accuracy than two-stage methods, which also makes it ideal for portable devices to improve the efficiency of relevant diagnosis on KOA. However, all current studies have been confined to processing regular images. The effectiveness of one-stage or two-stage methods in the application of complex image results still needs to be compared in future studies.

Additionally, this diagnostic model can be applied to these scenarios: (1) in outpatient services, it can assist doctors in clinical diagnosis and can be further used in telemedicine. (2) Patients can obtain a preliminary diagnosis about their current severity of KOA by uploading radiographs. (3) For surgeons who lack clinical experience, it can be used to assist learning and accumulate experience for their growth. (4) In the future, it can be combined with other algorithms to form a prediction model to assess a patient's disease progression and prognosis.

There are main advantages of this diagnostic model: (1) it achieves satisfactory accuracy in avoiding cumbersome image preprocessing; thus, users can take photos directly in an easy-to-acquired way; (2) the automatic assessment process requires high reliability and repeatability results, and the possible differences between manual diagnoses by surgeons are effectively avoided; and (3) it avoids interference from implants, markers in radiographs and some degree of angle changes of conventional shooting (some of the photos show unilateral prosthesis, internal or external fixation). When clicking around the knee joint on a screen to display the diagnostic model's K-L scale, if it detects impairment, there was no display around the knee.); (4) a direct aid to remote clinical decision-making; (5) a patient prioritization model that shortens waiting time and improves patient experience and satisfaction; and (6) a practical approach that facilitates medical education, training, and research. The diagnostic model has potential disadvantages. A machine occasionally requires software and hardware updates to meet the latest requirements, which may require costs. Artificial intelligence based on preloaded data and experience cannot be creative like surgeons.

This study has limitations. First, surgeon preference, experience, and ability may influence ascertaining the diagnoses and assessments [32]. Second, although the study shows that this model has good reliability and reproducibility in radiology assessment, the specific diagnosis still needs other evidence, such as the patient’s clinical manifestations, laboratory tests, and other imaging findings [12]. Third, in the process of use, due to the restrictions of the algorithm, there may be some photos in which the entire knee joints cannot be identified, and the diagnostic model does not display a diagnosis if the algorithm detects implants or no object. Therefore, there may be a subset that is not identified as implants or knee joints, which requires rephotographing or further diagnosis by surgeons. Fifth, compared to similar studies, our sample size is relatively small and from a single hospital, and outcomes from a larger cohort and multiple countries may be different. Sixth, we propose the deep-learning-based RefineDet for object detection and classification, but the outcomes may be different based on other algorithms.

## Conclusion

The deep learning-based diagnostic model can be used to assess the severity of KOA in portable devices. On the premise of improving the diagnostic efficiency, the results are highly reliable and reproducible.

## Data Availability

The datasets used or analyzed during the current study are available from the corresponding author on reasonable request.

## Abbreviations

KOA: Knee osteoarthritis
K-L: Kellgren–Lawrence
PA X-ray: Posterior–anterior view standing bearing X-ray
AUC: Area under curve
LAT: Lateral
ARM: Anchor refinement module
ODM: Object detection module
TCB: Transfer connection block
IoU: Intersection over Union
TKA: Total knee arthroplasty
UKA: Unicompartmental knee arthroplasty
I-F: Internal fixation
E–F: External fixation
PFA: Patellofemoral arthroplasty
CHD: Coronary heart disease
